# Supplementary figures and images for: Genome-wide identification of HSF family in peach and functional analysis of PpHSF5 involvement in root and aerial organ development
Source: PeerJ. 2021 Mar 12;9:e10961. doi: 10.7717/peerj.10961 (PMC7958895; doi:10.7717/peerj.10961)

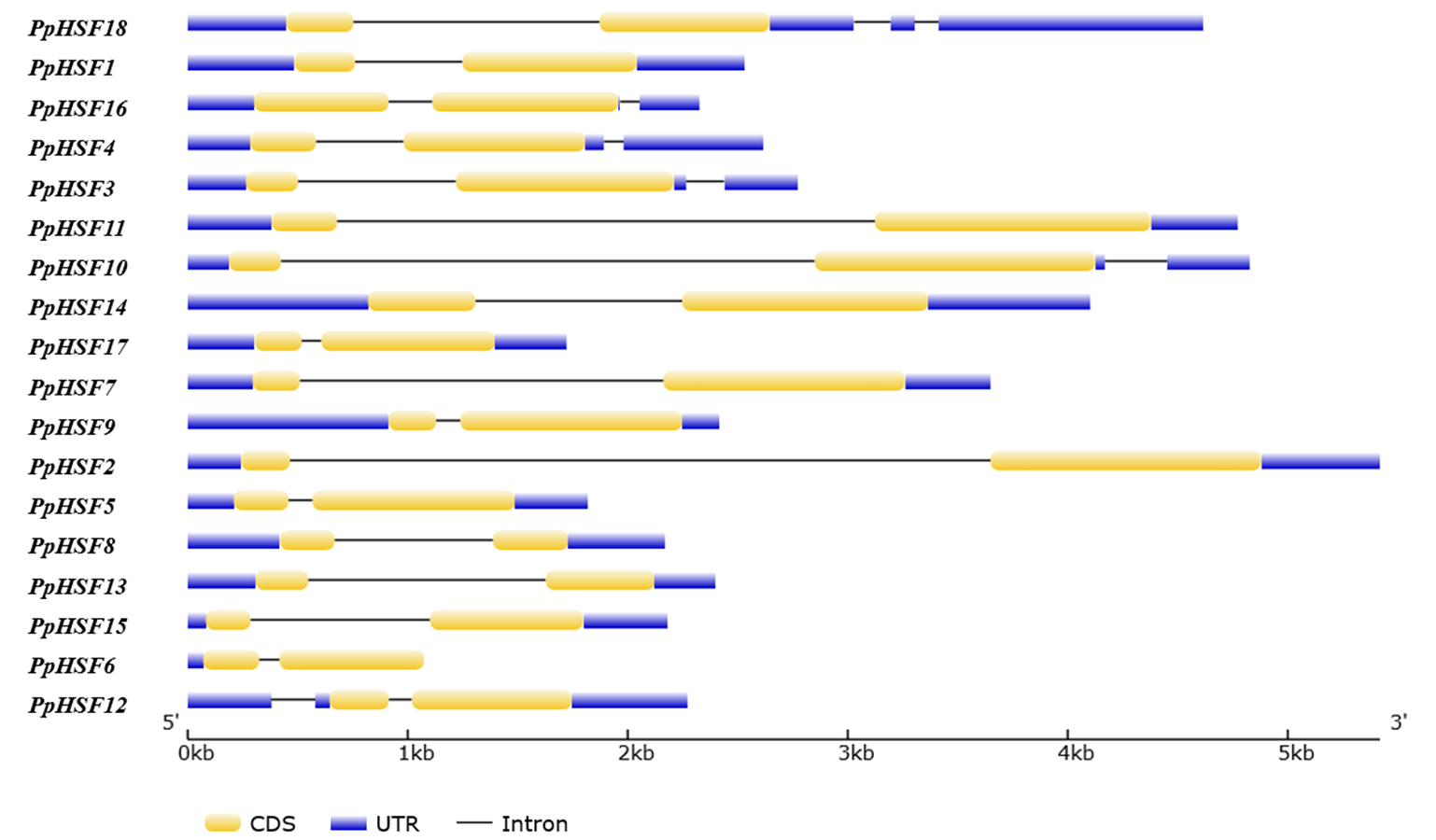

Supplement: Supplemental Information 9 — Predicted Coding Sequences (CDS) are in yellow, introns are a flat line, and the untranslated regions (UTRs) are shown in blue. [file peerj-09-10961-s009.png]
